# Differential Mechanism of ATP Production Occurs in Response to Succinylacetone in Colon Cancer Cells

**DOI:** 10.3390/molecules24193575

**Published:** 2019-10-03

**Authors:** Phil Jun Lee, Seung Je Woo, Hee Min Yoo, Namki Cho, Hong Pyo Kim

**Affiliations:** 1 College of Pharmacy and Research Institute of Pharmaceutical Science and Technology (RIPST), Ajou University, Suwon 443-749, Korea; dlvlfwns83117@hanmail.net (P.J.L.); vlfwnstnwjd@naver.com (S.J.W.); 2 Center for Bioanalysis, Korea Research Institute of Standards and Science (KRISS), Daejeon 34113, Korea; 3 College of Pharmacy, Chonnam National University, Gwangju 61186, Korea

**Keywords:** succinylacetone, colon cancer cell lines, apoptosis, ATP, oxidative phosphorylation, pyruvate dehydrogenase

## Abstract

Our aim was to verify the potential ability of succinylacetone (SA) to inhibit mitochondrial function, thereby suppressing cancer cell proliferation. SA treatment caused apoptosis in HCT116 and HT29 cells, but not in SW480 cells, with mitochondria playing a key role. We checked for dysfunctional mitochondria after SA treatment. Mitochondria of HT29 cells were swollen, indicating damage, whereas in HCT116 cells, several mitochondria had a diminished size. Damaged mitochondria decreased ATP production and induced reactive oxygen species (ROS) in the cells. To understand SA-induced reduction in ATP production, we investigated the electron transfer chains (ETC) and pyruvate dehydrogenase kinase (PDK) activity, which prevents the transfer of acetyl-CoA to the TCA (tricarboxylic acid) cycle by inhibiting PDH (pyruvate dehydrogenase) activity. In each cell line, the inhibitory mechanism of ATP by SA was different. The activity of complex III consisting of the mitochondrial ETCs in HT29 cells was decreased. In contrast, PDH activity in HCT116 cells was reduced. Nicotinamide nucleotide transhydrogenase (NNT)-removing reactive oxygen species (ROS) was upregulated in HT29 cells, but not in HCT116 cells, indicating that in HT29 cells, a defense mechanism was activated against ROS. Collectively, our study showed a differential mechanism occurs in response to SA in colon cancer cells.

## 1. Introduction

Succinylacetone (4,6-dioxoheptanoic acid, SA) belongs to a class of compounds known as medium-chain keto acids. SA acts as an inhibitor of the enzyme 6-aminolevulinic acid dehydratase and induces dysfunctional mitochondria through the inhibition of heme synthesis [1,2]. Based on this fact, many studies have used SA to verify the role of heme, which is essential for several key cellular functions. Interestingly, a study reported that it appears unlikely that growth inhibition of L1210 cells by SA can be ascribed to heme depletion [3]. The intracellular role of SA may be cell-specific.

Colon cancer is a leading cause of cancer-related deaths globally [4]. It is a multifaceted disease that includes genetic, environmental, dietary, gut physiology, and lifestyle risk factors and is the third most commonly diagnosed cancer in men and women. The main cause of colon cancer is alteration in metabolism of lipids such as fatty acids, polar lipids, oxylipins, and triacylglycerols. To treat colon cancer, a combination of chemotherapy drugs such as oxaliplatin, 5-fluorouracil, and irinotecan are often used. However, these therapies have side effects. A recent study has described that mitochondria-targeting drugs can be considered a potential therapy to overcome colon cancer development.

The mitochondrion is a double-membrane-bound organelle found in most eukaryotic organisms. It produces ATP, which is a complex organic chemical that provides energy to drive various processes in living cells. For ATP production, pyruvate is first produced by glycolysis in the cytosol. Next, to transport pyruvate from the cytosol to the mitochondrial matrix, pyruvate is catalyzed by pyruvate dehydrogenase (PDH). Therefore, the mitochondrial enzyme PDH must be strictly regulated. Pyruvate dehydrogenase complex (PDC) is a multi-enzyme that catalyzes the oxidative decarboxylation of pyruvate to produce acetyl-CoA. This complex consists of three enzymes: pyruvate dehydrogenase (E1), dihydrolipoyl transacetylase (E2), and dihydrolipoyl dehydrogenase (E3). The complex plays an important role in regulating glycolytic flux through carbohydrate oxidation and the rate of fatty acid oxidation [5]. Interestingly, the step after pyruvate production is determined according to the characterization of the cell. In the mitochondria of normal cells, electrons that are transferred through Complexes I, III, and IV extrude protons outward into the intermembrane space, generating a proton gradient that drives ATP synthase (Complex V), as protons pass back through the inner membrane into the matrix [6]. In contrast, cancer cells produce lactate with ATP production by consuming glucose (the Warburg effect). Interestingly, emerging evidence shows that oxidative phosphorylation occurs during ATP production in cancer cells [7].

A recent study showed that heme deficiency induced apoptosis in HeLa cells, a well-characterized cervical cancer cell line [8]. In line with this, because SA, an inhibitor of heme synthesis, decreases ATP production through mitochondrial dysfunction, SA may be used as a potential candidate to prevent cancer development. However, no study has yet reported development of any new anti-cancer therapy using SA. In our study, because elevated heme synthesis intensified oxidative metabolism and tumorigenic functions in colon cancer [9,10], we tested anti-cancer capacity of SA in colon cancer.

## 2. Results and Discussion

### 2.1. SA Effect on the Viability of Colon Cancer Cells

**Figure 1:**
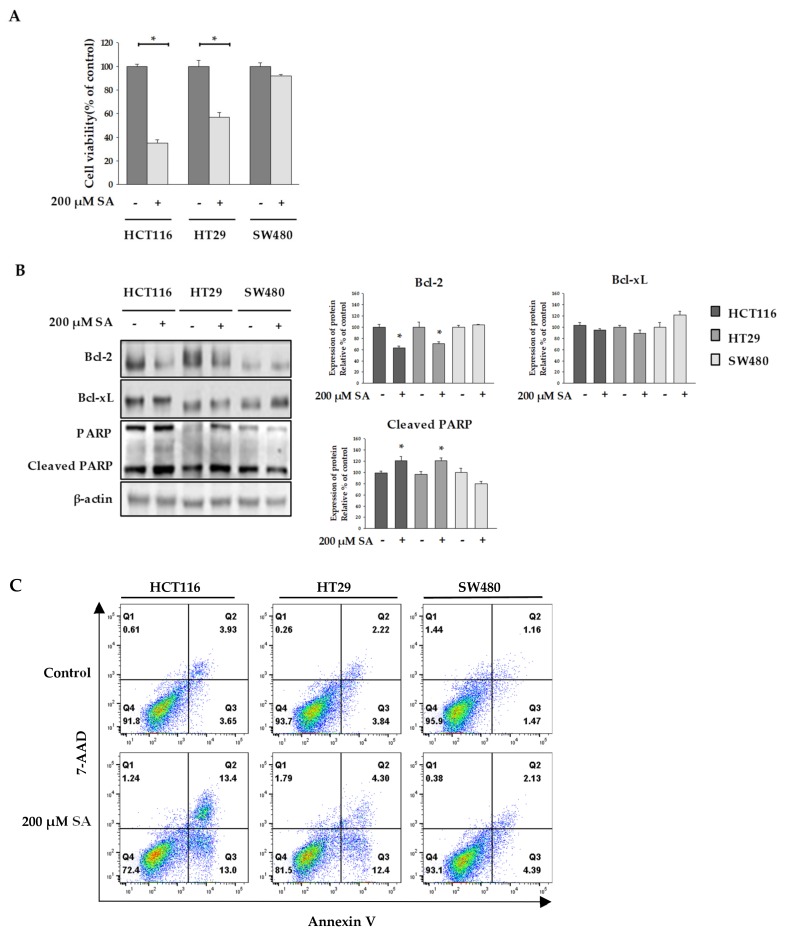
Effect of succinylacetone (SA) to viability of colon cancer cells. HCT116, HT29, and SW480 cells were treated with 200 µM SA for 48 h. (**A**) MTT assay was performed to assess cell viability. (**B**) Expression of Bcl-2, Bcl-xL, and cleaved-PARP was detected by Western blotting. β-actin was reprobed as a loading control. (**C**) Cells were incubated with 200 μM of SA for 48 h and subsequently stained with Annexin-V/PI. Data are representative values of three independent experiments and expressed as mean ± SD (*n* = 3), * *p* < 0.05.

In this study, we optimized the concentration of SA using the MTT assay. As shown in Figure 1A, cell viability of HCT116 and HT29 cells dramatically reduced, whereas no effect of SA in SW480 cells was seen. To differentiate the effect of 200 µM SA on selective cell death in colon cancer cell from its effect on normal cells, we examined the cytotoxicity of 200 µM SA in CCD18-Co (normal colon) and MRC-5 (normal lung). Treatment of SA up to 1mM did not lead to cell death in both normal cells (Figure S1). This observation suggested that SA selectively induces apoptosis in HCT116 and HT29 cells, but not in normal cells. In addition, expression levels of an anti-apoptotic protein, B-cell lymphoma 2 (Bcl-2) and B-cell lymphoma-extra large (Bcl-xL), and a pro-apoptotic protein, cleaved-Poly (ADP-ribose) polymerase (cleaved-PARP) were assessed to determine whether SA induced apoptosis [11]. We found that Bcl-2 expression decreased and that of cleaved-PARP increased in HCT116 and HT29 cells (Figure 1B). However, no robust changes were observed in the expression of Bcl-xL. In addition, flow cytometry analysis of damaged cells probed with Annexin V/7-AAD demonstrated that the cells underwent apoptosis following incubation with SA (Figure 1C). The lower right quadrant (Annexin-V+/PI−) and the upper right quadrant (Annexin-V+/PI+) of the figure presents the percentage of cells in early and late apoptosis, respectively. Thus, a single treatment with SA strongly induced apoptosis of HCT116 and HT29 cells, but not SW480 cells.

**Figure 2:**
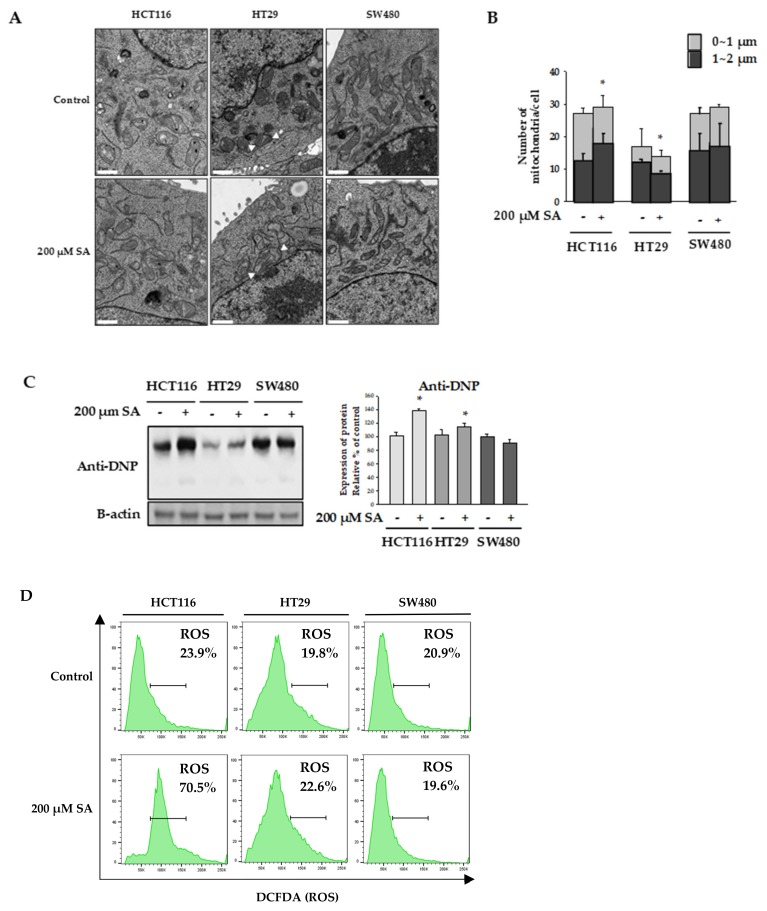
Mitochondrial morphology. HCT116, HT29, and SW480 cells were treated with 200 µM SA for 48 h. (**A**) The cells were fixed and viewed under a transmission electron microscope. Main observation is indicated by white arrows (scale bars: 1 μm). (**B**) Comparison of the number and size of mitochondria in each cell. (**C**,**D**) Intracellular ROS levels were measured using an anti-Dinitrophenol (anti-DNP) by Western blot and the 2′,7′-dichlorodihydrofluorescein diacetate (H2DCFDA). Data are representative values of three independent experiments and expressed as mean ± SD (*n* = 3), * *p* < 0.05.

Mitochondria play a key role in apoptotic processing. Many studies have shown that damaged mitochondria are swollen [12]. We observed mitochondrial morphology to check whether mitochondria were dysfunctional. Mitochondria of HT29 cells treated with SA were swollen compared to those in the control group (Figure 2A). In contrast, mitochondria in HCTT116 cells were smaller in size compared to those in the control group, and the number of small mitochondria increased with treatment of 200 μM SA for 48 h (Figure 2B). No significant difference was observed in SW480 cells.

Damaged mitochondria produce reactive oxygen species (ROS), which play a key role in cell viability [13]. A relationship between ROS production and apoptosis caused by anticancer agents has been demonstrated [13,14]. In our experiment, intracellular ROS levels were measured by western blotting using anti-dinitrophenol (anti-DNP), as well as flow cytometry with 2′,7′-dichlorodihydrofluorescein diacetate (H2DCFDA). We observed that SA treatment increased the expression of DNP in both HCT116 and HT29 cells. Consistent with this, flow cytometry results demonstrated that ROS generation in HCT116 cells was significantly increased by SA treatment, while the levels of ROS were not increased in SW480 cells (Figure 2D). Together, these findings suggest that ROS generation upon SA treatment is a potential mechanism underlying ROS-dependent apoptosis in HCT116 colon cancer cells.

### 2.2. SA Decreased ATP Levels through a Differential Mechanism

**Figure 3:**
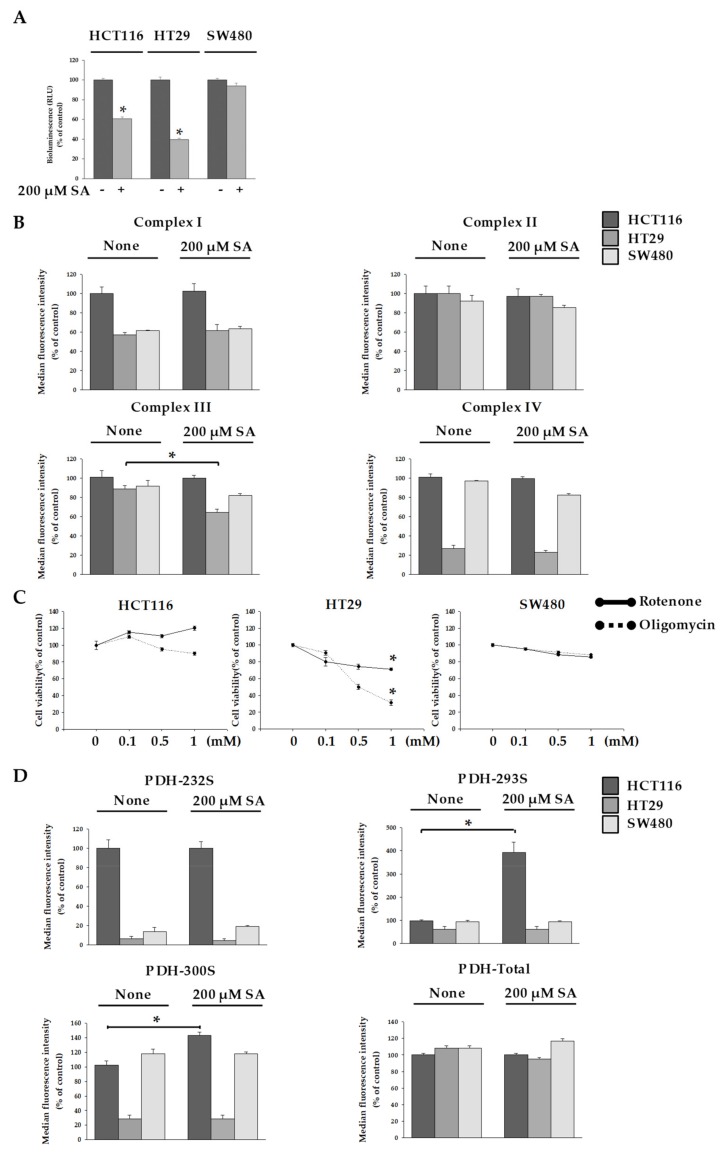
Measurement of electron transport chain (ETC) and pyruvate dehydrogenase (PDH) activity. HCT116, HT29, and SW480 cells were treated with 200 µM SA for 48 h. (**A**) ATP levels in cells treated with SA treatment compared to untreated control. (**B**) Activity of complexes in HCT116, HT29, and SW480 cells treated with SA was measured. (**C**) Each cell was treated with rotenone or oligomycin on various concentration-dependent for 48 h, and cell viability was analyzed. (**D**) pyruvate dehydrogenase kinase (PDK) levels in the cells were measured to confirm PDH activity. Data are representative values of three independent experiments and expressed as mean ± SD (*n* = 3), * *p* < 0.05.

Dysfunctional mitochondria resulted in the reduction of ATP levels [15]. We measured ATP levels in cells after SA treatment. The amount of ATP was decreased by approx. 40% in HCT116 cells and by approx. 60% in HT29 cells after SA treatment (Figure 3A). However, no change of ATP in SW480 cells was seen. 

Because ATP is the energy source of cells, apoptosis in HCT116 and HT29 cells after SA treatment was accompanied by a decrease in ATP levels. To gain insight into the ATP decrease by SA, we investigated the electron transfer chain (ETC) and pyruvate dehydrogenase kinase (PDK) activity, which prevents the transfer of acetyl-CoA to the tricarboxylic acid (TCA) cycle by inhibiting pyruvate dehydrogenase (PDH) activity. In general, collapse of the mitochondrial membrane potential results in decreased Complex I and Complex III activity. In addition, a recent study has reported that Complex IV activity decreases upon the collapse of mitochondrial membrane potential in hippocampal neurons of primary cultured rats and in human fibroblasts (IMR90) [16]. Therefore, we investigated the activity of Complex III, and the mitochondria from the electron transport chain in HT29 and HCT116 cells, and observed a significant decrease in the activity of Complex III in HT29 cells, but not HCT116 cells (Figure 3B). To further support our argument, we observed the cells after inhibiting ETC activity in mitochondria using oligomycin and rotenone [17]. We found that approx. 30% of HT29 cells survived after oligomycin treatment at the highest concentration used (1 mM), and approx. 60% survived after rotenone treatment at the highest concentration used (1 mM) (Figure 3C). Interestingly, despite decreased levels of ATP production, HCT116 cells exhibited no significant differences in mitochondrial ETC activity. This suggests that the 200 µM SA concentration in HCT116 cells does not affect mitochondrial activity, and instead decreases ATP production through a different mechanism. We focused on the previous step of TCA cycle. The mitochondrial enzyme PDH must be constantly regulated [18]. PDH is a multi-enzyme that catalyzes the oxidative decarboxylation of pyruvate to produce acetyl-CoA. Therefore, we measured the activity of pyruvate dehydrogenase kinase (PDK) to understand the mechanism of reduced ATP production in HCT116 cells due to SA. An increase in PDK activity inhibits PDH activity, which prevents acetyl-CoA from being transported to the TCA cycle [19]. PDH activity is inhibited when three specific positions (Ser232, Ser293, and Ser300) are phosphorylated by PDK [20]. Thus, we investigated the degree of phosphorylation of PDH (Ser232, Ser293, and Ser300). Results showed that phosphorylation occurred to a notable degree at Ser293 and Ser300 only in the case of HCT116 cells (Figure 3D). This signifies a decrease in PDH activity. Thus, SA acts as a PDK activity inducer which induced the concentration of acetyl-CoA in HCT116 cells with SA. No interesting result in SW480 cells with SA was seen. Additionally, we observed that apoptosis in SW480 cells did not occur in spite of the treatment of 1mM SA. Therefore, single treatment of SA seemed not to be suitable to inhibit SW480 cells.

### 2.3. Nicotinamide Nucleotide Transhydrogenase (NNT) Activity in HT29 Cells Abolished ROS Production

**Figure 4:**
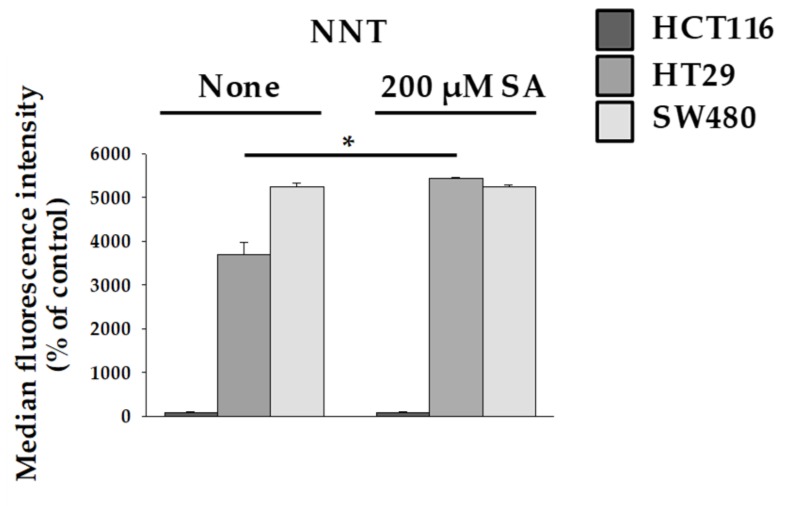
Measurement of nicotinamide nucleotide transhydrogenase (NNT) activity. HCT116, HT29, and SW480 were treated with 200 µM SA for 48 h. Data are representative values of three independent experiments and expressed as mean ± SD (*n* = 3), * *p* < 0.05.

Mitochondrial damage and disruption of the mitochondrial membrane leads to mitochondrion-dependent apoptosis through ROS production [21]. When the TCA cycle is unable to transport electrons through the ETC, intracellular ROS can be produced using reduced nicotinamide adenine dinucleotide (NADH) and reduced flavin adenine dinucleotide (FADH). The main production sites of ROS have been reported as Complex I and Complex III [22]. However, in our study, despite the decrease in Complex III levels in HT29 cells, the increase in ROS levels occurred to a lesser extent. To understand this result, we investigated the protective action of Nicotinamide Nucleotide Transhydrogenase (NNT). NNT is an integral protein of the inner mitochondrial membrane that uses the proton motive force generated by the ETC to catalyze the transfer of hydride (H-) from NADH to NADP+. About half of the mitochondrial NADPH pool is sensitive to uncouplers and is thus likely to be generated by NNT [23]. Interestingly, ROS are eliminated by enzymes that require NAPDH, which indicates that elevated NNT level inhibits ROS production. Our study showed that NNT activity increases in HT29 cells (Figure 4). This result is consistent with the finding that ROS production slightly increased in SA-treated HT29 (Figure 2C). Thus, it is possible that a protective mechanism is activated in HT29 cells to prevent ROS overproduction.

## 3. Materials and Methods

### 3.1. Cell Culture

Colon cancer cell lines HCT116, HT129, and SW480 were purchased from the Korean Cell Line Bank (KCLB, Seoul, South Korea). RPMI 1640 (Welgene, Seoul, South Korea) supplemented with 10% FBS and 1% penicillin/streptomycin (Gibco; Thermo Fisher Scientific, Waltham, MA, USA) was used as the culture medium. Cells were maintained in an incubator at 37 °C and 5% CO_2_. When cells cover at least 80% of the surface of the 100-mm cell culture dish, they were sub-cultured using a trypsin-EDTA solution.

### 3.2. Reagents

Succinylaceton, oligomycin, and rotenone were purchased from Sigma-Aldrich (St Louis, MO, USA).

### 3.3. Cell Viability Measurement (MTT Assay)

The MTT (3-(4,5-Dimethylthiazol-2-yl)-2,5-diphenyl tetrazolium bromide) assay was used to measure the proliferation-inhibiting activity of SA on HCT116, HT29, and SW480 cells. Each colon cancer cell line was placed in a 12-well-plate and cultured. After the medium was replaced with fresh DMEM, SA was added to the cells till a final concentration of 200 µM and cells were cultured for 48 h. Next, MTT solution was added to each well and allowed to react at 37 °C for 4 h. All media were removed, and formazan was dissolved by sufficiently shaking in dimethyl sulfoxide (DMSO). An ELISA microplate reader was then used to measure absorbance at 562 nm. The experiments were performed at least three times, with cells for each condition plated in triplicate. Further, the mean value and standard error were used as the final values.

### 3.4. Western Blotting

Colon cancer cells were treated with SA at a final concentration of 200 µM. Cells were cultured for 48 h and washed with cold phosphate buffer saline (PBS) and lysed with a radioimmunoprecipitation (RIPA) assay buffer containing 1× PBS, 1% (*v*/*v*) Nonidet P-40 (NP-40), 0.5% (*w*/*v*) sodium deoxycholate, 0.1% (*w*/*v*) sodium dodecyl sulfate (SDS), 0.1 mg/mL phenylmethylsulfonyl fluoride (PMSF), 30 μL/mL aprotinin and 1 mM sodium orthovanadate (Na2VO3). Cell lysates were centrifuged and the resulting supernatants collected. Proteins were separated on an 8–15% SDS–polyacrylamide gel and transferred to a polyvinylidene difluoride (PVDF) membrane. Membrane was blocked in Tris-buffered saline (TBS) containing 0.1% Tween 20 (TBST) and 5% non-fat dry milk for 1 h at room temperature and then incubated overnight with primary antibodies in TBST containing 1% non-fat dry milk at 4 °C. Anti- BCL-2, anti-BCL-xL, anti-PARP, and anti-*β*-actin were purchased from Santa Cruz Biotechnology (Dallas, TX, USA). Anti-cleaved PARP was purchased from Cell Signaling Technologies (Danvers, MA, USA). Anti-dinitrophenol was purchased from Abcam (Cambridge, MA, USA). Membranes were washed with TBST and incubated with goat, anti-rabbit, or anti-mouse horseradish peroxidase-conjugated IgG secondary antibody for 2 h. The signal was quantified using the chemiluminescence system (GE Healthcare, Piscataway, NJ, USA) [24]. Bands were quantified by densitometric analysis using Image J software. Data are representative of three independent experiments.

### 3.5. Transmission Electron Microscopy (TEM) Observation of Mitochondria

TEM was employed to observe the morphologies of the CRC cells affected by SA in greater detail. The three types of CRC cells were plated on 100-mm cell culture dishes and cultured for 48 h. Then, cells were treated with SA at a final concentration of 200 µM and were cultured for 48 h. After washing with cold PBS, trypsin-EDTA treatment was performed to detach the cells from the cell culture dish. Karnovsky’s fixative reagent was used for 2 h of primary fixation. After washing three times with 0.05 M sodium cacodylate buffer, secondary fixation was performed with 0.1 M cacodylate-buffer and 0.2% osmium tetroxide for 2 h. After dehydration with EtOH, the sample was firmly fixed with resin. Ultrathin sections were made and observed using a JEM-1010 (JEOL, Tokyo, Japan) microscope.

### 3.6. Measurement of PDH Activity Within Cells

The PDH kit containing magnetic beads (EMD Millipore; catalog No. PDHMAG-13K Kit, Burlington, MA, USA), which were provided as a 1 × stock, was used. Beads were sonicated for 30 s, then vortexed, and 25 μL was added to each well following the addition of 25 μL of Assay Buffer 1, and either 25 μL of lysate control or 25 μL of sample. The plate was incubated on a plate shaker protected from light for 2 h at room temperature. The well contents were removed and the plate was washed three times using a magnetic plate washer. Afterwards, 50 μL of detection antibody was added and the plate incubated on a plate shaker at room temperature for 1 h. Next, 50 μL SAPE was added to each well, and the plate incubated on a plate shaker at room temperature for 30 min. The beads were then resuspended in 100 μL Sheath Fluid. Finally, the plate was read using a Luminex 200™ system (Luminex, Austin, TX, USA) and analyzed using the accompanying Milliplex Analyst software (EMD Millipore, Billerica, MA, USA).

### 3.7. Measurement of Mitochondrial Electron Transport Chain Activity

The mitochondrial ETC activity in CRC cells was quantitatively analyzed using the Cellular Metabolism Multiplex Assay kit (Merck KGaA, Darmstadt, Germany). Experiments were conducted according to the protocols provided by the manufacturer.

### 3.8. Measurement of Intracellular ATP Concentration

Colon cancer cells were treated with SA at a final concentration of 200 µM and were cultured for 48 h. The cells were washed with cold PBS. Washed cells were centrifuged at 900× *g* for 10 min and the supernatant was discarded. RIPA buffer containing a protease inhibitor cocktail was added; 100 mM Tris, 4 mM EDTA, and a pH 7.75 buffer were subsequently added, each with volumes equal to nine times the amount of the RIPA buffer. The mixture was then mixed thoroughly and placed in a 100 °C incubator for 2 min. After centrifugation at 900× *g* for 1 min, the supernatant was mixed with a luciferase reagent. Afterwards, the amount of ATP was measured using the ATP Bioluminescence Assay Kit CLS II (Roche, Penzberg, Germany) [25].

### 3.9. Determination of SA-Induced Apoptosis

SA-treated HCT116, HT129, and SW480 were visualized by flow cytometry using the PE Annexin V Apoptosis Detection Kit with 7-AAD (BioLegend, San Diego, CA, USA). SA-treated and water treated (control) cells were seeded in 6-well plates and incubated at 37 °C for 48 h. Cells were harvested 48 h post-treatment, washed with phosphate-buffered saline (PBS), and resuspended in binding buffer (1×). Cells were stained with annexin V-PE and 7-AAD, per the manufacturer’s instructions. After staining, the cells were discriminated as early (Annexin V^+^/7-AAD^–^) and late (Annexin V^+^/7-AAD^+^) apoptotic cells using a flow cytometer (BD FACSVerse, BD Biosciences, San Diego, CA, USA). Cell percentages were analyzed using the FlowJo software (TreeStar, Ashland, OR, USA).

### 3.10. Measuring Reactive Oxygen Species (ROS)

Intracellular ROS levels were detected using 2′,7′-dichlorodihydrofluorescein diacetate acetyl ester (H2DCFDA; Thermo Fisher Scientific, Waltham, MA, USA). For this analysis, cells were incubated with DCFDA (1 μM) for 15 min at room temperature. Cells were also washed with cold PBS and resuspended in 0.5 mL of PBS supplemented with 1% fetal bovine serum. Intracellular fluorescence accumulation was analyzed using a flow cytometer (BD FACSVerse, BD Biosciences, San Diego, CA, USA).

### 3.11. Statistical Analysis

Statistical analysis was performed by using the Student’s *t*-test to determine change in value compared to the control value. Results were considered statistically significant when the *p*-value was less than 0.05.

## 4. Conclusions

In this study, we investigated a differential mechanism which occurs in response to SA in colon cancer cells. SA induced apoptosis in HCT116 and HT29 cells, but not in SW480 cells. To uncover the mechanisms by which SA treatment induces apoptosis in HCT116 and HT29 cells, our study measured ATP and ROS levels. Cancer cells can produce ATP through aerobic glycolysis or oxidative phosphorylation. We confirmed that SA in both HCT116 and HT29 cells deceased ATP by regulating different mechanisms including the activity of complex III or PDK. In addition, ROS was significantly increased in HCT116 cells suggesting that apoptosis of HCT116 cells might be partially accompanied by elevated ROS levels. Our findings suggest that SA mediated inhibition of ATP production was cell-type dependent.

## Supplementary Materials

The following are available online, Figure S1: Effect of SA to viability of colon cancer cells and normal cells. (A-B) colon cancer cells (HCT116, HT29, and SW480) were treated with various concentrations. Likewise, normal cells (CCD-180o and MRC5) was incubated with SA under same condition. MTT assay was performed to assess cell viability. Data are representative values of three independent experiments and expressed as mean ± SD (*n* = 3), *** *p* < 0.005 compared to the control group.

## References

[1] Sassa S., Kappas A. (1983). Hereditary tyrosinemia and the heme biosynthetic pathway. Profound inhibition of delta-aminolevulinic acid dehydratase activity by succinylacetone. J. Clin. Invest..

[2] Huang M.L., Chiang S., Kalinowski D.S., Bae D.H., Sahni S., Richardson D.R. (2019). The role of the antioxidant response in mitochondrial dysfunction in degenerative diseases: cross-talk between antioxidant defense, autophagy, and apoptosis. Oxid. Med. Cell Longev..

[3] Weinbach E.C., Ebert P.S. (1985). Effects of succinylacetone on growth and respiration of L1210 leukemia cells. Cancer Lett..

[4] Bhandari A., Woodhouse M., Gupta S. (2017). Colorectal cancer is a leading cause of cancer incidence and mortality among adults younger than 50 years in the USA: A SEER-based analysis with comparison to other young-onset cancers. J. Investig. Med..

[5] Gray L.R., Tompkins S.C., Taylor E.B. (2014). Regulation of pyruvate metabolism and human disease. Cell Mol. Life Sci..

[6] Mourier A., Larsson N.G. (2011). Tracing the trail of protons through complex I of the mitochondrial respiratory chain. PLoS Biol..

[7] Kim S.Y. (2018). Cancer energy metabolism: Shutting power off cancer factory. Biomol. Ther. (Seoul).

[8] Ye W., Zhang L. (2004). Heme deficiency causes apoptosis but does not increase ROS generation in HeLa cells. Biochem. Biophys. Res. Commun..

[9] Kabe Y., Nakane T., Koike I., Yamamoto T., Sugiura Y., Harada E., Sugase K., Shimamura T., Ohmura M., Muraoka K. (2016). Hame-dependent dimerization of PGRMC1/Sigma-2 receptor facilitates cancer proliferation and chemoresistance. Nat. Commun..

[10] Hooda J., Cadinu D., Alam M.M., Shah A., Cao T.M., Sullivan L.A., Brekken R., Zhang L. (2013). Enhanced heme function and mitochondrial respiration promote the progression of lung cancer cells. PloS ONE.

[11] Lee P.J., Shin I., Seo S.Y., Kim H., Kim H.P. (2014). Upregulation of both heme oxygenase-1 and ATPase inhibitory factor 1 renders tumoricidal activity by synthetic flavonoids via depleting cellular ATP. Bioorg. Med. Chem. Lett..

[12] Zorov D.B., Juhaszova M., Sollott S.J. (2014). Mitochondrial reactive oxygen species (ROS) and ROS-induced ROS release. Physiol. Rev..

[13] Redza-Dutordoir M., Averill-Bates D.A. (2016). Activation of apoptosis signalling pathways by reactive oxygen species. Biochim. Biophys. Acta..

[14] Yu T., Robotham J.L., Yoon Y. (2006). Increased production of reactive oxygen species in hyperglycemic conditions requires dynamic change of mitochondrial morphology. Proc. Natl. Acad. Sci. USA.

[15] Cogliati S., Enriquez J.A., Scorrano L. (2016). Mitochondrial cristae: Where beauty meets functionality. Trends Biochem. Sci..

[16] Atamna H., Newberry J., Erlitzki R., Schultz C.S., Ames B.N. (2007). Biotin deficiency inhibits heme synthesis and impairs mitochondria in human lung fibroblasts. J. Nutr..

[17] Wyatt C.N., Buckler K.J. (2004). The effect of mitochondrial inhibitors on membrane currents in isolated neonatal rat carotid body type I cells. J. Physiol..

[18] Koukourakis M.I., Giatromanolaki A., Sivridis E., Gatter K.C., Harris A.L. (2005). Pyruvate dehydrogenase and pyruvate dehydrogenase kinase expression in non small cell lung cancer and tumor-associated stroma. Neoplasia.

[19] Park S., Jeon J.H., Min B.K., Ha C.M., Thoudam T., Park B.Y., Lee I.K. (2018). Role of the pyruvate dehydrogenase complex in metabolic remodeling: Differential pyruvate dehydrogenase complex functions in metabolism. Diabetes Metab. J..

[20] Hou X., Zhang L., Han L., Ge J., Ma R., Zhang X., Moley K., Schedl T., Wang Q. (2015). Differing roles of pyruvate dehydrogenase kinases during mouse oocyte maturation. J. Cell Sci..

[21] Pieczenik S.R., Neustadt J. (2007). Mitochondrial dysfunction and molecular pathways of disease. Exp. Mol. Pathol..

[22] Hagopian K., Weber K.L., Hwee D.T., Van Eenennaam A.L., López-Lluch G., Villalba J.M., Burón I., Navas P., German J.B., Watkins S.M. (2010). Complex I-associated hydrogen peroxide production is decreased and electron transport chain enzyme activities are altered in n-3 enriched fat-1 mice. PLoS ONE.

[23] Ciccarese F., Ciminale V. (2017). Escaping Death: Mitochondrial Redox Homeostasis in Cancer Cells. Front Oncol..

[24] Lee P.J., Woo S.J., Jee J.G., Sung S.H., Kim H.P. (2015). Bisdemethoxycurcumin induces apoptosis in activated hepatic stellate cells via cannabinoid receptor 2. Molecules.

[25] Lee P.J., Park H.J., Cho N., Kim H.P. (2018). 3,5-Diethoxy-3′-hydroxyresveratrol (DEHR) ameliorates liver fibrosis via caveolin-1 activation in hepatic stellate Cells and in a mouse model of bile duct ligation injury. Molecules.

