# Supplementary material for: Differential Mechanism of ATP Production Occurs in Response to Succinylacetone in Colon Cancer Cells
Source: Molecules. 2019 Oct 3;24(19):3575. doi: 10.3390/molecules24193575 (PMC6803967; doi:10.3390/molecules24193575)
Supplement: Supplementary file 1 [file molecules-24-03575-s001.pdf]

molecules

MDPI

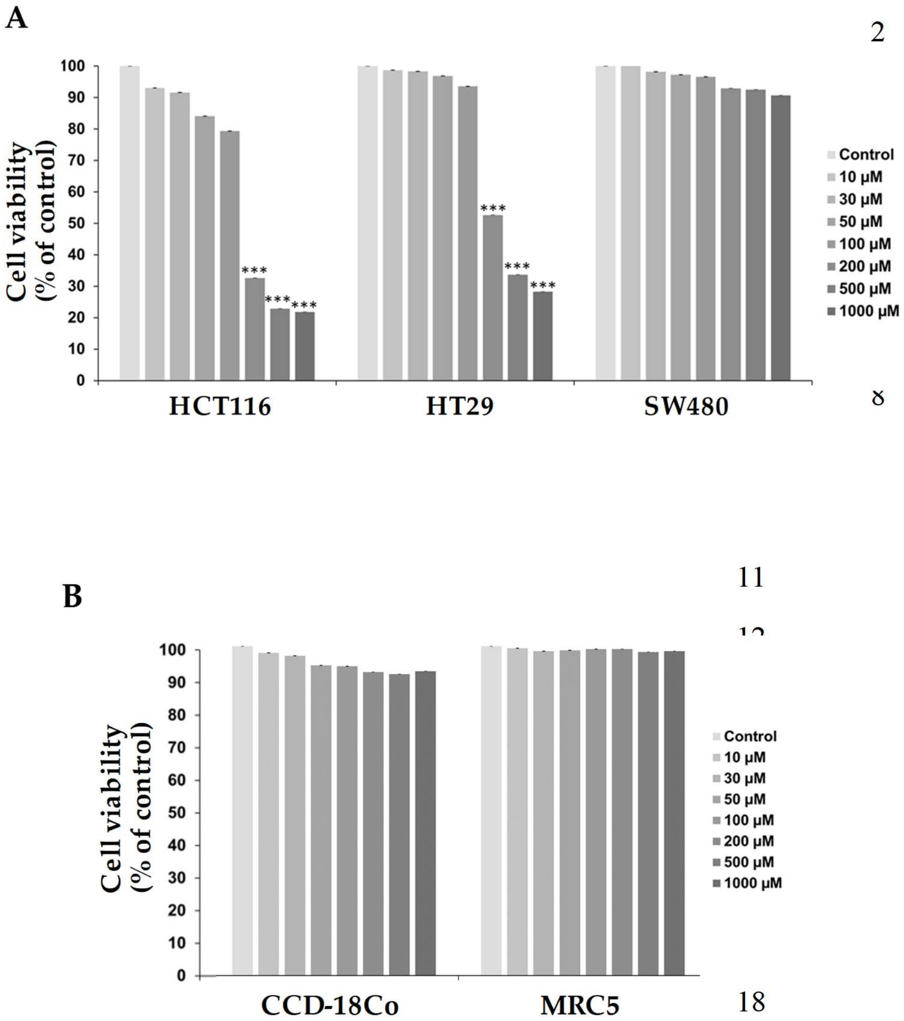

Figure 1S. Effect of SA to viability of colon cancer cells and normal cells. (A-B) colon cancer cells (HCT116, HT29, and SW480) were treated with various concentrations. Likewise, normal cells (CCD-18Co and MRC5) was incubated with SA under same condition. MTT assay was performed to assess cell viability. Data are representative values of three independent experiments and expressed as mean ± SD (n = 3), ***p<0.005 compared to the control group.
